# Irrigation has a higher impact on soil bacterial abundance, diversity and composition than nitrogen fertilization

**DOI:** 10.1038/s41598-021-96234-6

**Published:** 2021-08-19

**Authors:** Haoran Li, Hongguang Wang, Bin Jia, Dongxiao Li, Qin Fang, Ruiqi Li

**Affiliations:** State Key Laboratory of North China Crop Improvement and Regulation/Key Laboratory of Crop Growth Regulation of Hebei Province, College of Agronomy, Hebei Agricultural University, 2596#, Lekai South street, Baoding, 071000 Hebei China

**Keywords:** Environmental microbiology, Soil microbiology

## Abstract

The aim of this study was to assess the effects of irrigation frequency and nitrogen fertilization rate on the abundance, diversity, and composition of soil bacteria in winter wheat. Irrigation, but not nitrogen fertilization, significantly affected the bacterial alpha diversity index. Among the 50 phyla obtained in these treatments, Proteobacteria, Bacteroidetes, Actinobacteria, Acidobacteria, Gemmatimonadetes, and Firmicutes were the predominant phyla. The LEfSe analysis of different treatments indicated that irrigation had a stronger effect on soil bacteria community composition than nitrogen fertilization. Moreover, the soil pH, moisture, available phosphorus (AP), and available potassium (AK) significantly correlated with the relative abundance of dominant bacteria at the phylum, genus, and operational taxonomic unit (OTU) levels. Overall, after three years of irrigation and fertilization treatments, the effect of irrigation on soil bacteria abundance, diversity, and composition of winter wheat was stronger than that of nitrogen fertilization, highlighting the importance of water availability for bacteria communities in semi-arid ecosystems. Inorganic and organic fertilizers should be applied in rotation.

## Introduction

During the growing season of winter wheat, a large amount of fertilizer and groundwater irrigation are needed, resulting in groundwater pollution and a continuous decline in groundwater level, which causes the North China Plain to face a severe water resource crisis^1^. The North China Plain is an important grain production base in China, which has a long history of wheat–maize rotation agricultural system^2^. Water shortage is clearly a limiting factor for sustainable agricultural development in the North China Plain and other semi-arid areas. Wheat is one of the most important crops globally^3^. Therefore, it is important to study the effects of nitrogen fertilizer combined with irrigation on the quantity, diversity, and community structure of soil microorganisms.

The microbial community is an essential component of the soil and plays an important role in maintaining the ecological functions of the soil. It is directly involved in nutrient cycling, energy flow, and degradation of organic matter^4^. The microbial community is an important index for evaluating the health and quality of the soil. Soil microbial communities are very sensitive to soil changes, such as disturbances due to land tillage and irrigation water^5^. Maintaining the complexity and diversity of soil microbial communities is critical to the sustenance of soil fertility because soil microbes mediate the biogeochemical cycles of carbon and nitrogen, and serve as important reservoirs for plant nutrients^6^. Fertilization and irrigation are the main agricultural management measures in agricultural production that can promote crop growth and increase yield, and it can also affect soil microbial community^7^. Fertilization does not only provide the necessary nutrients for plant growth but also improves the characteristics of the soil due to the improvement in the nutrient utilization rate^8^. He et al. reported that long-term fertilization changed soil pH, total carbon, total nitrogen, and available phosphorus, which may lead to significant differences in microorganisms^8^. However, Yang et al. found that fertilization did not significantly change the composition and structure of the bacterial community, and the effect on bacterial diversity was not significant, because fertilization only increased the content of organic carbon and did not affect the pH value^9^. In this study, nitrogen fertilizer was used to clarify the mechanism of the effect on microorganisms.

Nitrogen is an essential element that plays a crucial role in natural and managed ecosystems, including agro-ecosystems, where it is often a common limiting factor in plant productivity and yield^10^. Nitrogen fertilizer plays an irreplaceable role in fertilization, therefore, rational and efficient application is particularly important. Nitrogen fertilization has been shown to alleviate the negative effects of plant-soil interaction^11^. Nitrogen availability largely impacts microbial communities^12^. Zeng et al. observed a change in the soil community structure and a decrease in bacteria diversity^13^. Fierer et al. found that nitrogen fertilization may directly or indirectly lead to changes in major microbial life-story strategies, which is conducive to a more active and copiotrophic microbial community^14^. This pattern is similar to the frequently observed K-selected plants being replaced by r-selected plants, while the nitrogen value of r-selected plants increased.

Yuan et al. found that the effect of irrigation water-type on soil microbial community structure was greater than that of nitrogen fertilizer treatment^15^. Soil irrigation management also affects the quantity, community structure, and diversity of soil microorganisms^16^. Soil water content changes the composition and activity of soil microbial communities through its effect on the transport of soil nutrients, substrate availability, and other soil properties^17^. Scarcity of water reduces soil organic carbon, thereby altering the soil microbial community structure^18^. Bai et al. found that moderate water supplementation, especially at the tasseling, postulation, and maturity stages, promotes maize growth and the diversity and composition of related rhizosphere microorganisms^19^. Moreover, the richness and evenness of bacteria increased with an increase in the soil relative water content. Soil microorganisms are important for the availability of underground nitrogen, and many processes of soil organic matter decomposition, mineralization, and biological nitrogen fixation driven by soil microorganisms are affected by nitrogen and water availability^20^. Hence, water and nitrogen availability interactively affect the soil microbial community and agricultural ecosystem functioning. The distribution of soil bacteria in natural and agricultural ecosystems has been extensively studied^21^. Bacterial community diversity and activity are considered more sensitive than other biota in response to changes in soil conditions such as pH, organic carbon, and nitrogen status^22^. Therefore, we will consider bacteria as the object of this study to evaluate their responses to different treatments.

In recent years, with the rapid development in DNA sequencing technology, next-generation sequencing (high-throughput sequencing) has been widely applied in the study of microbial communities. Next-generation sequencing technology has the advantages of a deep sequencing degree and large amount of data, which can reveal the complexity and diversity of the microbial community more accurately, and accelerate the research of non-culturable and trace microorganisms in the environment^23^. Previous studies have focused on the effects of single practices, such as nitrogen fertilization or irrigation, on soil bacterial communities. There have been few systematic studies on the effects of the combined management of nitrogen fertilization and irrigation on soil bacteria communities^24^. In this study, IonS5 sequencing was used to determine the soil bacteria community structure, diversity, and abundance in the Xinji Experimental Station, following three years of nitrogen fertilization and irrigation. Based on the data analysis, we can explore the influence of nitrogen fertilizer and irrigation on the bacteria community of wheat soil and provide a basis for optimizing the system of nitrogen fertilizer and irrigation, thereby improving soil fertility, maintaining soil microbial ecosystems, and achieving sustainable agricultural development in the North China Plain. Based on the results of previous studies, we hypothesized that nitrogen fertilizer and irrigation would affect the soil bacteria community composition and diversity by increasing or decreasing the relative abundance of specific bacteria classifications. In addition, we hypothesized that nitrogen fertilizer and irrigation would improve soil fertility by affecting soil chemical properties.

## Results

### Soil chemical characteristics and grain yields

After three years of conducting the experiment, the irrigation frequency was observed to have had significant effects on pH (p < 0.01), moisture (p < 0.001), total nitrogen (TN) (p < 0.001), C/N ratio (p < 0.001), and grain yield of winter wheat (p < 0.01), but not on available phosphorus (AP), available potassium (AK), or bulk density (BD). With increasing irrigation frequency, the pH decreased significantly, but moisture significantly increased (Table 1). TN was significantly higher under W0 (no irrigation) than under W1 (irrigation once at jointing) and W2 (irrigation twice at jointing and anthesis). The C/N ratio was significantly higher under W1 than under W0. Grain yield was significantly higher under W1 and W2 than under W0. Nitrogen fertilization rate had significant effects on TN (p < 0.001), SOC (p < 0.001), C/N ratio (p < 0.001), and grain yield (p < 0.001), but not on pH, moisture, AP, AK, or BD. With the increase in nitrogen fertilization rate, TN increased significantly, but the C/N ratio significantly decreased. The SOC and grain yield of winter wheat were significantly higher under N1 (nitrogen fertilization rate: 120 kg ha^−1^) and N2 (nitrogen fertilization rate: 240 kg ha^−1^) than under N0 (nitrogen fertilization rate: 0 kg ha^−1^). The interaction between irrigation frequency and nitrogen fertilization rate had no significant effect on any of the soil variables.

**Table 1:** Effect of different treatments and the interaction between irrigation frequency and nitrogen fertilization rate on soil chemical characteristics and grain yield of winter wheat.

Treatment	pH	Moisture (%)	TN (g kg^−1^)	SOC (g kg^−1^)	C/N ratio	AP (mg kg^−1^)	AK (mg kg^−1^)	BD (g cm^−3^)	Yield (kg ha^−1^)
W0N0	7.87 ± 0.03	6.75 ± 1.33	0.57 ± 0.01	16.96 ± 0.67	17.27 ± 0.78	36.83 ± 0.59	164.22 ± 7.36	1.20 ± 0.01	4819.40 ± 422.78
W0N1	7.91 ± 0.00	6.32 ± 1.08	0.73 ± 0.01	18.44 ± 0.11	14.59 ± 0.08	36.73 ± 0.89	163.75 ± 1.94	1.22 ± 0.01	5460.93 ± 239.69
W0N2	7.90 ± 0.05	7.51 ± 0.78	0.97 ± 0.04	18.91 ± 0.47	11.31 ± 0.23	36.98 ± 0.22	166.24 ± 5.32	1.20 ± 0.02	5769.62 ± 345.46
W1N0	7.78 ± 0.03	9.92 ± 0.33	0.50 ± 0.02	16.80 ± 0.63	19.38 ± 0.78	37.06 ± 0.15	165.64 ± 4.35	1.23 ± 0.02	5167.18 ± 174.49
W1N1	7.77 ± 0.01	7.93 ± 0.20	0.71 ± 0.02	18.33 ± 0.18	14.91 ± 0.55	36.88 ± 0.05	167.44 ± 4.03	1.22 ± 0.01	6561.62 ± 91.55
W1N2	7.79 ± 0.04	8.54 ± 0.51	0.92 ± 0.03	18.66 ± 0.64	11.77 ± 0.18	37.06 ± 0.26	169.61 ± 6.72	1.20 ± 0.01	6545.36 ± 434.36
W2N0	7.76 ± 0.03	12.39 ± 0.53	0.54 ± 0.02	16.78 ± 0.64	18.14 ± 0.16	37.03 ± 0.17	169.83 ± 7.74	1.21 ± 0.01	5299.40 ± 242.69
W2N1	7.75 ± 0.02	12.06 ± 1.12	0.71 ± 0.04	18.41 ± 0.07	14.99 ± 0.68	37.04 ± 0.14	169.53 ± 6.86	1.22 ± 0.02	6639.02 ± 260.63
W2N2	7.73 ± 0.03	11.11 ± 1.65	0.91 ± 0.04	18.77 ± 0.45	11.98 ± 0.67	37.04 ± 0.20	166.86 ± 7.04	1.22 ± 0.01	6776.07 ± 57.83
**Irrigation frequency**									
W0	7.89 ± 0.03a	6.86 ± 1.08c	0.76 ± 0.18a	18.10 ± 0.97a	14.39 ± 2.61b	36.84 ± 0.56a	164.74 ± 4.78a	1.21 ± 0.01a	5349.98 ± 514.89b
W1	7.78 ± 0.03b	8.80 ± 0.94b	0.71 ± 0.18b	17.93 ± 0.97a	15.35 ± 3.35a	37.00 ± 0.18a	167.56 ± 4.80a	1.22 ± 0.02a	6091.39 ± 733.07a
W2	7.75 ± 0.03c	11.85 ± 1.18a	0.72 ± 0.16b	17.99 ± 1.00a	15.04 ± 2.71ab	37.04 ± 0.15a	168.74 ± 6.41a	1.22 ± 0.01a	6238.16 ± 729.23a
**Nitrogen fertilization rate**									
N0	7.81 ± 0.06a	9.69 ± 2.56a	0.54 ± 0.03c	16.85 ± 0.57b	18.26 ± 1.07a	36.98 ± 0.34a	166.57 ± 6.30a	1.22 ± 0.02a	5095.33 ± 336.34b
N1	7.81 ± 0.07a	8.77 ± 2.68a	0.72 ± 0.02b	18.39 ± 0.12a	14.83 ± 0.48b	36.88 ± 0.47a	166.91 ± 4.82a	1.22 ± 0.01a	6220.53 ± 599.26a
N2	7.80 ± 0.08a	9.05 ± 1.86a	0.93 ± 0.14a	18.78 ± 0.47a	11.69 ± 0.47c	37.02 ± 0.20a	167.57 ± 5.76a	1.21 ± 0.01a	6363.68 ± 535.10a
**Two-way ANOVA**									
W	**	***	***	NS	***	NS	NS	NS	**
N	NS	NS	***	***	***	NS	NS	NS	***
W × N	NS	NS	NS	NS	NS	NS	NS	NS	NS

NS, not significant (p > 0.05).

*p < 0.05 significant levels.

**p < 0.01 significant levels.

***p < 0.001 significant levels.

Different letters within the same column denote significant differences (p < 0.05) among soils.

### Sequencing results and diversity indices

In total, we obtained 2,875,721 high-quality sequences from all 27 samples, and 102,258–110,990 sequences were obtained per sample with an average of 106,508. The read lengths ranged from 220 to 260 bp (mean = 252 bp). The values of the Good's coverage estimators were in the range of 0.982 and 0.985 at a 97% similarity cutoff, which suggested that the current numbers of the sequence reads were sufficient to capture the diversity of the soil bacteria community. When grouped at the 97% similarity level, there were a total of 11,360 OTUs in the complete data set (Table 2).

**Table 2:** The number of different bacteria taxon in the 27 soil samples.

Taxon	Phylum	Class	Order	Family	Genus	Species	OTU
Number	50	137	282	551	1242	2737	11,360

As shown in Table 3, irrigation frequency had a significant effect on richness (Sobs and chao1) (p < 0.05) and diversity (Shannon and PD) (p < 0.05), but not on evenness (Shannon even). Richness and diversity were significantly higher under W1 and W2 than under W0. Nitrogen fertilization rate and the interaction between irrigation frequency and nitrogen fertilization rate had no significant effect on the alpha diversity of bacteria.

**Table 3:** Effect of different treatments and the interaction between irrigation frequency and nitrogen fertilization rate on the richness, evenness and diversity of the bacteria community.

Treatment	Richness		Diversity		Evenness
	Sobs	Chao1	Shannon	PD	Shannon even
W0N1	5504 ± 343	6763 ± 273	6.70 ± 0.65	330 ± 16	0.843 ± 0.025
W0N2	5671 ± 150	6987 ± 150	7.19 ± 0.05	338 ± 10	0.828 ± 0.007
W1N0	5962 ± 213	7189 ± 223	7.37 ± 0.25	362 ± 7	0.847 ± 0.027
W1N1	6079 ± 161	7317 ± 323	7.51 ± 0.01	365 ± 5	0.862 ± 0.001
W1N2	6055 ± 156	7360 ± 134	7.50 ± 0.05	363 ± 4	0.862 ± 0.004
W2N0	6121 ± 249	7489 ± 330	7.47 ± 0.09	364 ± 21	0.856 ± 0.007
W2N1	5996 ± 260	7266 ± 168	7.44 ± 0.11	367 ± 16	0.859 ± 0.009
W2N2	6113 ± 271	7422 ± 309	7.40 ± 0.14	366 ± 8	0.849 ± 0.012
**Irrigation frequency**					
W0	5699 ± 266b	6968 ± 240b	7.01 ± 0.40b	340 ± 14b	0.841 ± 0.020a
W1	6032 ± 164a	7289 ± 221a	7.46 ± 0.15a	363 ± 5a	0.857 ± 0.015a
W2	6076 ± 233a	7392 ± 260a	7.44 ± 0.10a	366 ± 14a	0.854 ± 0.009a
**Nitrogen fertilization rate**					
N0	6002 ± 193a	7278 ± 263a	7.32 ± 0.22a	359 ± 13a	0.854 ± 0.016a
N1	5860 ± 354a	7115 ± 350a	7.22 ± 0.51a	354 ± 22a	0.831 ± 0.055a
N2	5946 ± 271a	7256 ± 275a	7.37 ± 0.16a	356 ± 15a	0.846 ± 0.016a
**Two-way ANOVA**					
W	*	*	*	*	NS
N	NS	NS	NS	NS	NS
W × N	NS	NS	NS	NS	NS

NS, not significant (p > 0.05).

*p < 0.05 significant levels.

**p < 0.01 significant levels.

***p < 0.001 significant levels.

Different letters within the same column denote significant differences (p < 0.05) among the soil samples.

### Phylogenetic diversity of bacteria communities

**Figure 1:**
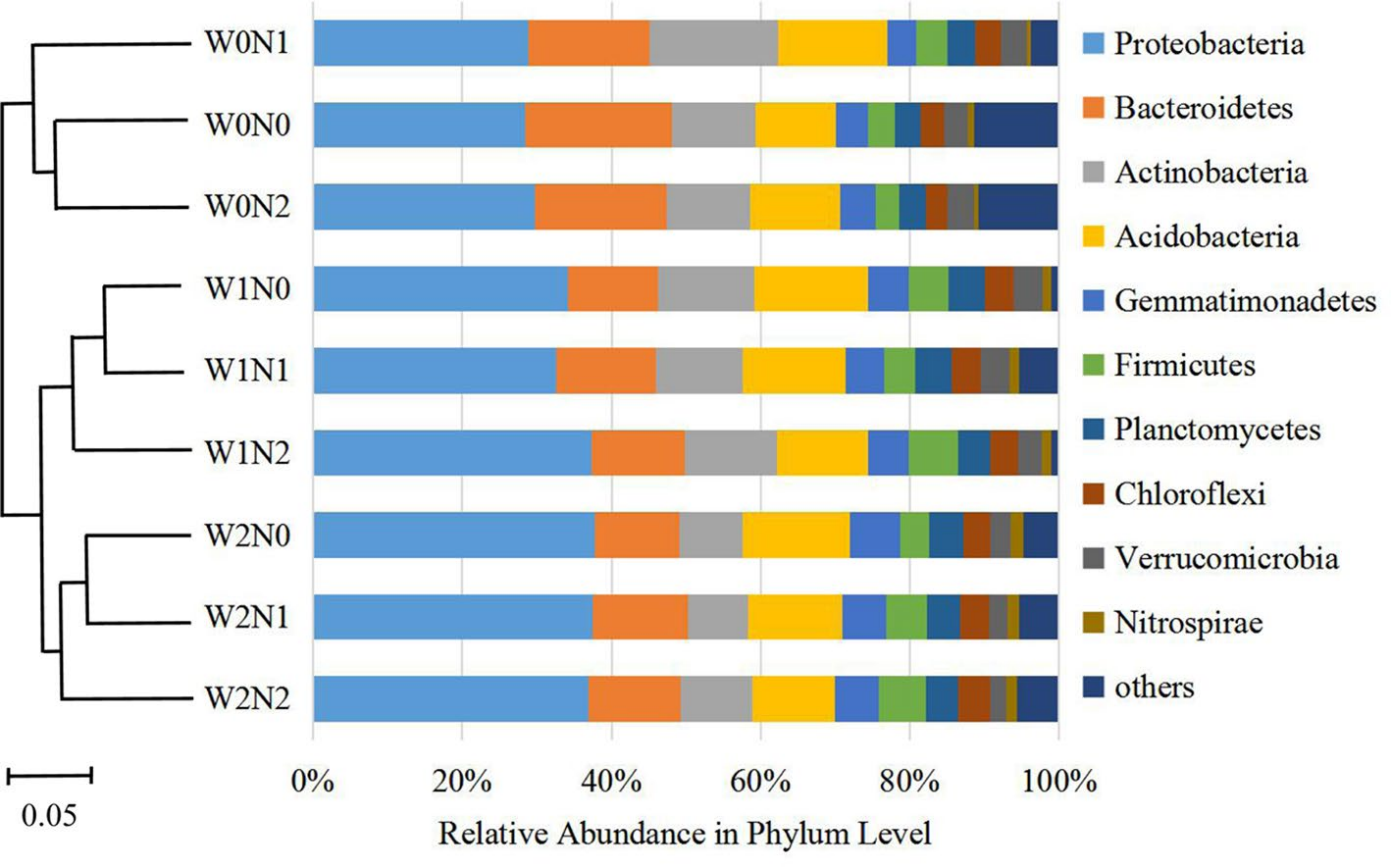
The relative abundance of major taxonomic groups at the phylum level for bacteria in different treatments of irrigation frequency and nitrogen fertilization rate, with the clustering tree on the left showing the similarities among treatments.

The relative abundance of dominant bacteria phyla across all the soil samples is shown on the right side of Fig. 1. The dominant bacteria phyla were the same across all treatments, but their relative abundance in soil samples varied: Proteobacteria (28.46–37.78%), Bacteroidetes (11.36–19.65%), Actinobacteria (8.01–17.27%), Acidobacteria (10.76–15.30%), Gemmatimonadetes (3.84–6.74%), Firmicutes (3.13–6.6%), Planctomycetes (3.41–4.96%), Chloroflexi (2.90–4.34%), Verrucomicrobia (2.24–3.81%), and Nitrospirae (0.64–1.68%). On the left side of Fig. 1, cluster analyses showed the relationships among the different treatments.

**Figure 2:**
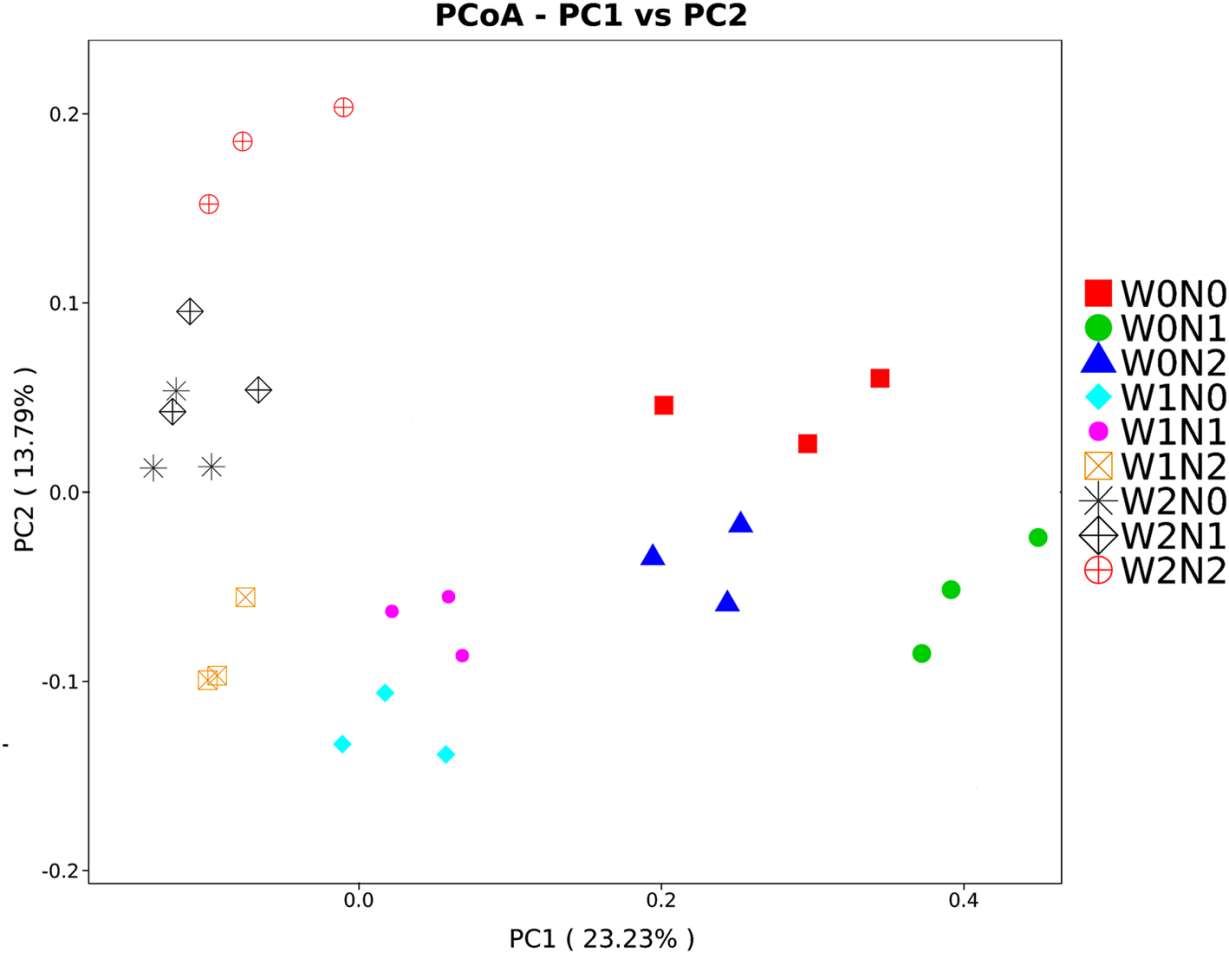
Principal coordinate analysis (PCoA) of soil bacteria community composition in different treatments of irrigation frequency and nitrogen fertilization rate. PCoA was calculated using the Bary-Curtis distance matrix.

PCoA analysis of soil samples explained 37.02% of the variation in bacterial community composition along the two axes (Fig. 2). The three replicates of irrigation and nitrogen fertilization treatments usually clustered closely, indicating the reproducibility of the profiles of the bacteria community. The different irrigation frequency treatments were significantly different from each other. Irrigation significantly affected the bacteria community composition of soils (PERMANOVA: F = 4.1107, p < 0.001), whereas no significant effects in nitrogen fertilization were observed (PERMANOVA: F = 0.6528, p = 0.758).

### Taxonomic composition

We conducted LEfSe analysis to identify the statistical significance of differentially abundant bacteria taxa and the biological relevance of the species in different treatments. Supplementary Figure S1 and Supplementary Fig. S2, and Supplementary Fig. S3 show the taxa abundance at different irrigation frequencies under the same N fertilization rate. When the nitrogen fertilization rate was 0, the phylum Gemmatimonadetes was more abundant in the soils of W2 than in the other treatments (Supplementary Fig. S1). Irrigation also significantly increased the abundance of the bacterial orders Xanthomonadales, Gemmatimonadales, Deltaproteobacteria, Gemmatimonadetes, OM190, and family Gemmatimonadaceae (Supplementary Fig. S1). At a nitrogen fertilization rate of 120 kg ha^−1^, the phylum Proteobacteria, class Gammaproteobacteria, and order Xanthomonadales were more abundant in the soils of W2 than in the other treatments (Supplementary Fig. S2). The order Pseudonocardiales was more abundant in the soils of W0 than in the other treatments (Supplementary Fig. S2). At nitrogen fertilization rate of 240 kg ha^−1^, the phyla Saccharibacteria, class Bacteroidia, order Bacteroidales, family Porphyromonadaceae, and genus *Adhaeribacter* were more abundant in the soils of W0 than in other treatments (Supplementary Fig. S3). The class Deltaproteobacteria was more abundant in the soils of W1 than in the other treatments (Supplementary Fig. S3). The phylum Nitrospirae, class Nitrospira, and genus *Nitrospira* were more abundant in the soils of W2 than in the other treatments (Supplementary Fig. S3).

Supplementary Figure S4 Supplementary Fig. S5, and Supplementary Fig. S6 show the taxa abundance at different nitrogen fertilization rates under the same irrigation frequency. At the irrigation frequency of 0, the classes Clostridia, Clostridiales, Porphyromonadaceae, Bacteroidales-S24-7-group, genera *Parabacteroides*, and *Schlesneria* were more abundant in the N0 soil than in the other treatments (Supplementary Fig. S4). In contrast, at the irrigation frequency of 1, the order Xanthomonadales, family Xanthomonadaceae, and genus *Lysobacter* were more abundant in the soil of N2 than in the other treatments (Supplementary Fig. S5). The family CHAB-XI-27 was more abundant in the soils of N1 than in the other treatments (Supplementary Fig. S5). However, at the irrigation frequency of 2, the abundance of bacteria in different treatments of nitrogen fertilization rate was not significantly different (Supplementary Fig. S6).

**Figure 3:**
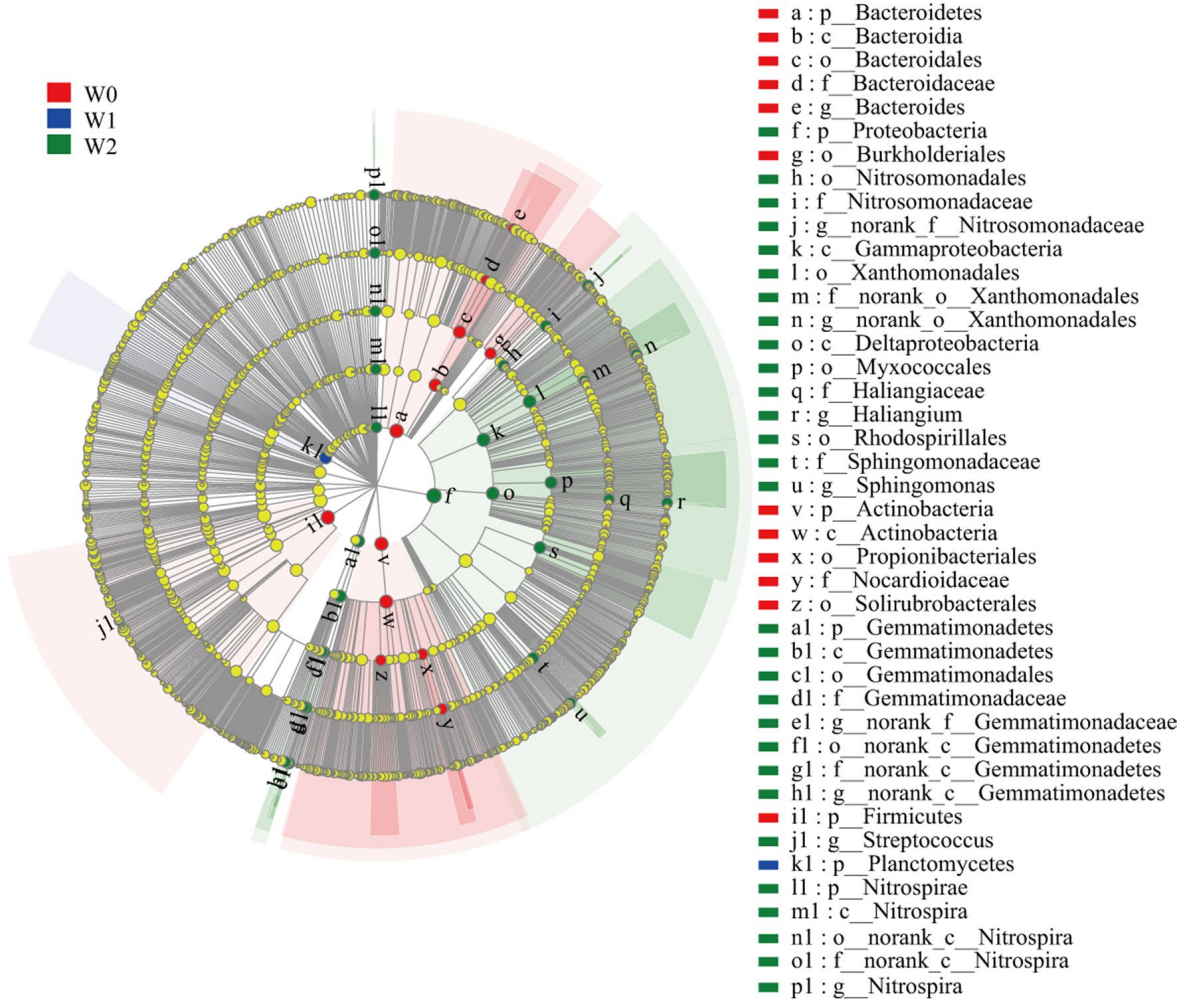
A linear discriminant analysis effect size (LEfSe) method identifies the significantly different abundant taxa of bacteria in different treatments of irrigation frequency (irrespective of nitrogen fertilization rate). The taxa with the absolute LDA > 3.5 and p < 0.05 are shown. This figure is drawn by LEfSe (Version 1.0, http://huttenhower.sph.harvard.edu/galaxy/root?tool_id=lefse_upload) software.

**Figure 4:**
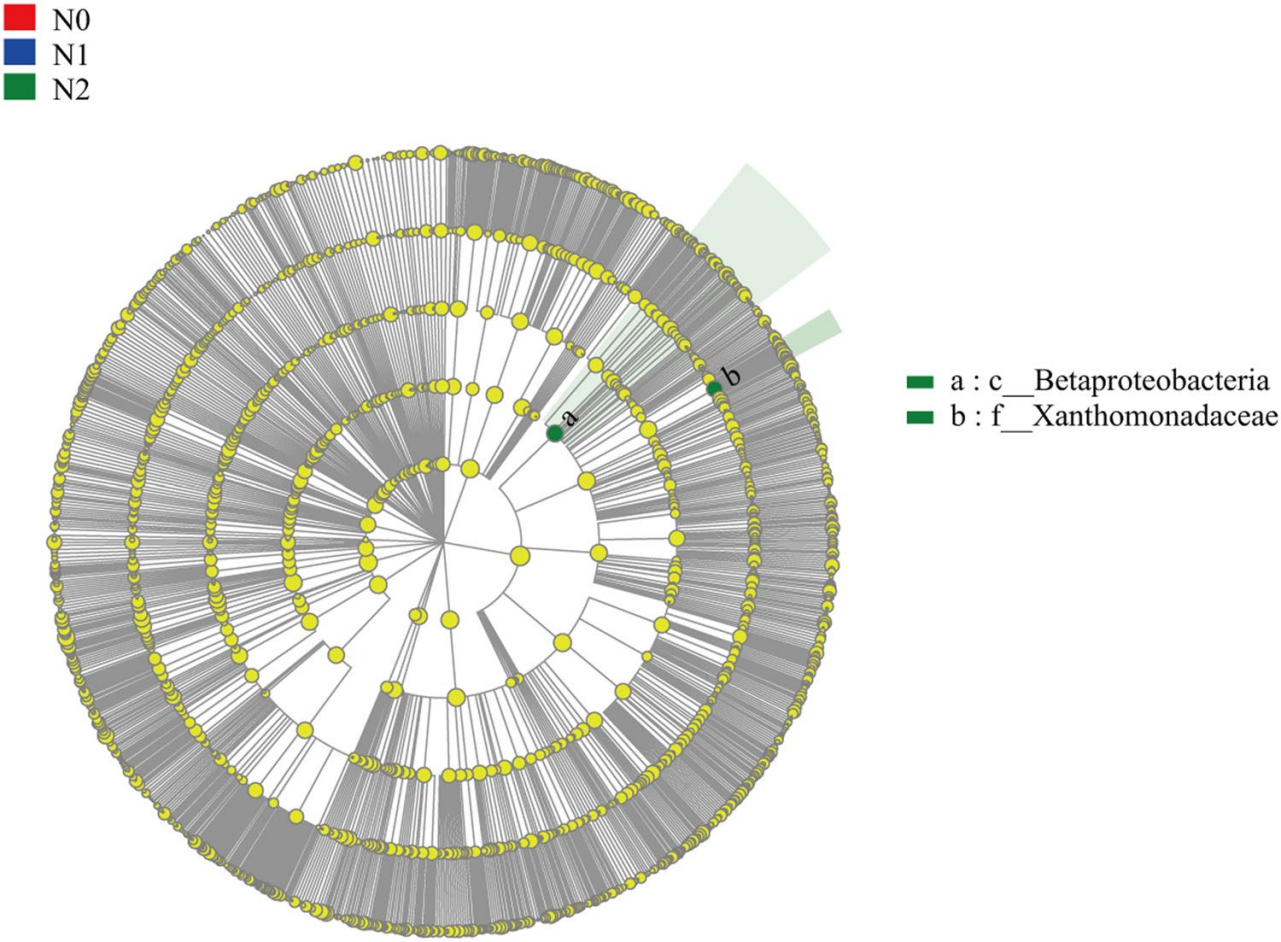
A linear discriminant analysis effect size (LEfSe) method identifies the significantly different abundant taxa of bacteria in different treatments of nitrogen fertilization rate (irrespective of irrigation frequency). The taxa with the absolute LDA > 3.5 and p < 0.05 are shown. This figure is drawn by LEfSe (Version 1.0, http://huttenhower.sph.harvard.edu/galaxy/root?tool_id=lefse_upload) software.

The taxa abundance resulting from different irrigation frequencies (irrespective of nitrogen fertilization rate) and nitrogen fertilization rates (irrespective of irrigation frequency) are shown in Figs. 3 and 4. The phyla Proteobacteria, Gemmatimonadetes, and Nitrospirae were more abundant in the soils of W2 than in the other treatments, whereas Bacteroidetes, Actinobacteria, and Firmicutes were more abundant in the soils of W0 (Fig. 3). Only phylum Planctomycetes was found to be more abundant in W1 than in the other treatments (Fig. 3). The class Betaproteobacteria and family Xanthomonadaceae were more abundant in the soils of N2 (Fig. 4). In general, irrigation significantly influenced the abundance of many bacteria, but nitrogen fertilization had no significant effect on bacteria (Fig. 4).

At the OTU level, 11,360 OTUs were identified in all the soils (Supplementary Table S1). Among the 10 most abundant OTUs, six OTUs were identified as dominantly enriched with a relative abundance > 0.5% in at least one sample. The six OTUs included OTU82 (norank-c-Acidobacteria), OTU95 (*Thermomonas*), OTU1428 (*RB41*), OTU1740 (norank-f-Gemmatimonadaceae), OTU2822 (norank-f-Nitrosomonadaceae), and OTU2894 (norank-c-Gemmatimonadetes). Irrigation frequency had significant effects on eight OTUs, but not on OTU82 (norank-c-Acidobacteria) or OTU6495 (norank-o-Subgroup-7). With the increase in irrigation frequency, OTU1428 (*RB41*) decreased significantly, whereas the other OTUs significantly increased. Nitrogen fertilization had no significant effect on the 10 OTUs (p > 0.05). The interaction between irrigation frequency and nitrogen fertilization rate had significant effects on OTU95 (*Thermomonas*) (p < 0.01), OTU1428 (*RB41*) (p < 0.05), OTU2822 (norank-f-Nitrosomonadaceae) (p < 0.01), and OTU6387 (norank-o-Xanthomonadales) (p < 0.001), but not on other OTUs.

### Correlations between alpha diversity, relative abundance of dominant bacteria, and soil properties

There was a significant negative correlation between pH and moisture content (Supplementary Table S2). The total nitrogen (TN) and soil organic carbon (SOC) were significantly and negatively correlated with the C/N ratio. There was a significant positive correlation between TN and SOC. SOC was significantly and positively correlated with the yield of winter wheat, whereas the C/N ratio and yield were negatively correlated.

The Pearson correlations between alpha diversity and soil properties showed that AP and AK were significantly and positively correlated with alpha diversity, whereas the correlation between pH and alpha diversity was significant and negative (Supplementary Table S2). Moisture was positively correlated with alpha diversity, but it was significantly correlated only with chao1 and PD. The correlations between the other indices and alpha diversity were not significant.

The Pearson correlations between the relative abundance of dominant bacteria and soil properties confirmed that pH, moisture, AP, and AK were the dominant factors correlating with bacterial community structures (Supplementary Table S2). The correlations between several soil properties and the relative abundance of many dominant bacteria were significant. At the phylum level, Firmicutes and Chloroflexi were significantly and positively correlated with the yield of winter wheat. Additionally, at the OTU level, OTU6387 (norank-o-Xanthomonadales) was significantly and positively correlated with yield.

## Discussion

Soil pH is the main driving force shaping soil microbial community^25^. Soil moisture content is also the main factor affecting soil microbial composition and activity^26^. The pH and moisture of the soil were mainly affected by irrigation, but not by nitrogen fertilizer (Table 1). Similar results were observed by Ding et al., who thought that the significant decrease in pH value in paddy soil might result from the increase in proton concentration in the soil solution due to the accumulation of organic matter under irrigation^27^. However, the correlation between pH and soil organic carbon (SOC) was not significant in this study, whereas the correlation between pH and moisture was negative and significant (Supplementary Table S2). This is probably because irrigation causes more nitrates and other negative ions to dissolve in the soil, which reduces soil pH^28^. Zhong et al. observed that the soil pH showed no significant differences among different nitrogen fertilization treatments, which is similar to our study^24^. Inconsistencies have also been reported in other studies that soil pH decreased with increased nitrogen fertilization^29^. It is likely that three years of nitrogen fertilization may still be too short to affect soil pH. Soil total nitrogen (TN) increased with increasing nitrogen fertilization and decreased with increasing irrigation. Higher soil moisture can foster microbial growth and promote metabolic activities^30^, which increases the efficiency of nitrogen utilization, thus lowering TN. Compared with W0N0, irrigation and nitrogen fertilization significantly increased the yield of winter wheat. However, the difference between W2 and W1 was not significant, as was the case with N1 and N2, which was consistent with the results of Wang et al.^31^ who found no significant relationship between crop yield and bacterial community, but a greater relationship with soil organic carbon, which is fully illustrated in this study (Supplementary Table S2).

Microbial diversity plays a key role in ecosystem stability and soil productivity by regulating soil chemical processes^32^, which it is essential for the sustainable development of agriculture^33^. Two-way ANOVA analysis of alpha diversity revealed the effects of irrigation and nitrogen fertilization (Table 3). Irrigation had a significant effect on alpha diversity, but nitrogen fertilization did not. As mentioned above, it is likely that irrigation significantly reduced the pH of the soil, but nitrogen fertilization did not. Peralta et al. reported that moderate wetting could improve the diversity of soil bacterial community structure, while excessive drying and wetting could do the opposite^34^. Some studies have found that water quantity is an important factor affecting microbial community diversity, and it was more important than land management or type of fertilizer^35^. The correlation between pH and alpha diversity was significant and negative (Supplementary Table S2). When the soil pH was close to neutral, the alpha diversity of the bacterial community was the highest^36^. These results indicate that a neutral environment is suitable for the survival of microorganisms. There may be two major effects of pH on bacteria. First, PH directly restricts the physiological process of soil bacteria, and if the soil pH value exceeds a certain range, it will reduce the growth of individual groups that cannot survive. Second, soil pH may not directly change bacterial diversity and community structure, but as a composite variable, the composite index providing soil conditions has many soil characteristics that are often directly or indirectly related to soil pH and may drive observed changes in diversity and community composition. In recent years, pH has been extensively studied in various soils as a major factor in determining the diversity and composition of soil bacteria^36^. Long-term nitrogen fertilization has been shown to increase microbial diversity^37^; however, no significant change may be observed in the short term over several years^38^, mainly because there was no change in pH, which was consistent with our study. Moreover, abundant nutrients can promote the growth of some bacteria^39^, which may explain why available phosphorus (AP) and available potassium (AK) were significantly and positively correlated with alpha diversity (Supplementary Table S2).

Soil microorganisms are the core component of terrestrial ecosystems. Their composition and structure can reflect, not only the efficiency of biological transformation, but also the state of soil fertility^40^. In this study, the Ion S5™ XL sequencing platform was used to study the structure of soil bacteria communities in nine treatments of nitrogen fertilization and irrigation. Fifty phyla, 137 classes, and 1242 genera were identified (Table 2). The results show that the dominant phyla in the different treatments were similar, but the relative abundances of the dominant phyla in the different treatments were different (Fig. 1). In agreement with previous studies^41^, the dominant phyla in each treatment were Proteobacteria, Bacteroidetes, Actinobacteria, Acidobacteria, and Gemmatimonadetes. Different nitrogen fertilization and irrigation treatments may alter the physicochemical properties of soil, which are often closely related to soil bacterial communities. Our results show that the soil chemical characteristics had different effects on the relative abundance of bacteria at different taxonomic levels. For example, the phylum, Nitrospirae, was not significantly correlated with soil AP, but the genus, Norank-f-Nitrosomonadaceae, was significantly and positively correlated with soil AP. Consistent with our study, Shen et al. observed that nitrogen fertilization and irrigation could alter soil bacterial community structure, but the effects of nitrogen fertilization and irrigation on bacteria community structure were different (Figs. 1 and 2)^42^. A similar community structure was found in the treatments with the same irrigation frequency, which indicated that the effects of irrigation on soil bacterial community structure were more significant than those of nitrogen fertilization.

The results of the different treatments indicated that irrigation had a stronger effect on soil bacteria community composition than nitrogen fertilization in this study (Supplementary Table S1, Fig. 3, and Fig. 4). The relative abundance of phyla Proteobacteria, Gemmatimonadetes, and Nitrospirae were higher in the W2 treatment, whereas the relative abundances of Bacteroidetes, Actinobacteria, and Firmicutes were higher in the W0 treatment (Fig. 3). Although other studies have shown similar results, this is still remarkable because all bacteria phyla are phylogenetic, and there could still be wide metabolic differences among them, making some bacteria phyla dominant in the soil, while others are always quite rare. Proteobacteria is a gram-negative bacterium that is highly susceptible to environmental disturbance and water-limiting stress^43^. Consistent with the results of our study, Dennis et al. found that the relative abundance of Proteobacteria increased with an increase in the irrigation amount^44^. This is because increasing soil moisture is beneficial for crop growth and metabolism, which promotes the secretion of some rhizomatous sediments. Proteobacterium can synthesize carbohydrates and proteins from the secretions of crops. Gemmatimonadetes were associated with phosphorus metabolism^45^, which showed a significant positive correlation with AP (Supplementary Table S2). Nitrospirae contain nitrite-oxidizing bacteria that are capable of nitrification and are part of the biogeochemical nitrogen cycle^46^. The correlation between the relative abundance of Bacteroidetes and Actinobacteria and soil pH was positive and has been observed in a previous study^47^, which is consistent with our study (Supplementary Table S2). Bacteroidetes play an important role in degrading cellulose in soils^48^. The relative abundance of Actinobacteria was strongly associated with long-term drought. Barnard et al. found that the relative abundance of Actinobacteria in grasslands decreased with increased irrigation^49^. Ammonifying and nitrifying bacteria play important roles in soil nitrogen metabolism. The common ammonifying bacteria in soils are *Pseudomonas*, *Bacillus*, *Clostridium*, *Arthrobacter*, and *Proteus*. In this study, there were significant differences in the abundance of some bacteria among the different treatments, but not in the ammonifying bacteria (Fig. 3 and Fig. 4). We found that short-term nitrogen application did not affect soil ammoniation, in contrast to previous research results^50^. Ammonifying bacteria are not particularly sensitive to environmental changes, and small changes in temperature, water content, and pH are do not affect their activity. Compared with ammonifying bacteria, nitrifying bacteria are more sensitive to changes in the soil environment. There were significant differences in the presence of nitrifying bacteria among the different irrigation treatments (Fig. 3 and Fig. 4). The abundance of *Nitrospira* in W2 was significantly higher than that in W0. *Nitrospira* can oxidize ammonia to nitrite, which is key to controlling the nitrification rate. Fan et al. found that the abundance of *Nitrospira* would increase with an increase in nitrogen application rate, which is a more accepted view at present^51^. However, irrigation had a significant effect on the abundance of *Nitrospira* in this study, and there were no significant differences between the different nitrogen fertilization treatments. As nitrate easily dissolves in water, excessive irrigation will cause nitrate loss, and nitrate leaching caused by irrigation is more serious than nitrogen application^52^. We speculated that nitrate leaching led to the reduction of nitrifying bacteria reaction products, which promoted the increase of *Nitrospira* by feedback to ensure the absorption of nitrogen by wheat roots. Due to the ability of wheat to absorb nitrogen and water, the structure and composition of soil microbial communities are affected by intensive agricultural practices.

In summary, our results suggest that most changes in bacteria community parameters were influenced by soil water and pH, which were significantly correlated with irrigation levels, while nitrogen fertilizer had no significant effect on bacteria. Long-term application of inorganic fertilizer shows that nitrogen fertilizer could effectively increase crop yield, but also significantly reduce bacterial diversity. However, some studies have indicated that the application of nitrogen fertilizer has little or no effect on bacteria communities, especially in a short time^53^. This may be due to the fact that although some harmful local conditions appeared after urea application, the short-term effect of fertilization on soil microbial community was minimal. The application of urea and other nitrogen fertilizers resulted in a high concentration of ammonia nitrogen in the local crop soil, which may have a strong inhibition or killing effect on microorganisms. However, it should be noted that the harsh environment caused by nitrogenous fertilizers is generally limited in space. Although studies have shown that concentrations of ammonium ions in long-term fertilized soil may be higher than those in unfertilized soil. Concentrations at most times and places, in the short term, may be well below levels that are toxic to microorganisms^54^. Under laboratory culture conditions, approximately 200 kg N was applied to urea as a nitrogen fertilizer. After 10 days, the microbial community composition changed significantly; however, after 90 days, the microbial community returned to its original level^55^. The above results show that the effect of nitrogen fertilizer would be more significant after long-term application. The effect of irrigation on bacteria communities may be more pronounced than that of nitrogen fertilization. Some studies have emphasized that drought inhibits microbial activity, which in turn affects soil fertility-related microorganisms and impedes their mediated biogeochemical processes^56^. Genomic analysis showed that under the condition of reduced irrigation, the decomposition rate of organic matter slowed down, which was related to the change in community structure^57^. The influence of relative soil water concentration on the microbial community composition was significant. We speculate that reducing irrigation will reduce soil enzyme activity and have a long-term impact on soil nutrient availability and plant supply, and thus have a significant impact on crop yield. Based on the results from this and previous studies, it can be concluded that increased water availability due to irrigation treatment has important implications for microbial communities, soil function, and crop production in semi-arid ecosystems.

Water and nitrogen interacted with each other, and irrigation and nitrogen fertilization at higher levels had a significant inhibitory effect on other management measures. Water dissolved all kinds of nutrients, including nitrogen, and sufficient soil water indicate efficient nutrient supply. High irrigation diluted the high concentration of ammonia nitrogen caused by local nitrogen fertilization, so that nitrogen was evenly distributed in the crops. However, too much nitrogen had irreversible effects, permanently affecting the bacteria and even causing death. This feedback between irrigation and nitrogen fertilization is shown in Supplementary Fig. S4, Supplementary Fig. S5, and Supplementary Fig. S6. In the absence of irrigation, nitrogen fertilizer did not dissolve sufficiently and was likely to accumulate and cause bacterial damage, and this effect was more significant with the increase in nitrogen fertilization rate (Supplementary Fig. S4). Irrigation significantly altered this toxic effect (Supplementary Fig. S6). When irrigated in large quantities, the effect of water on bacteria was much greater than that of nitrogen fertilizer, resulting in no significant difference between different nitrogen treatments (Supplementary Fig. S6).

## Conclusion

This study shows that water is the key factor affecting the abundance, diversity, and composition of soil bacteria. Our results suggest that most changes in bacteria community parameters were influenced by soil pH, which is significantly correlates with irrigation levels. Soil pH and moisture were significantly correlated with the relative abundance of dominant bacteria at the phylum, genus, and OTU levels. Irrigation affects the composition of bacteria communities by changing the abundance of certain bacteria, such as Proteobacteria, Gemmatimonadetes, Nitrospirae, Bacteroidetes, Actinobacteria, and Firmicutes. This could serve as basis for optimizing the system of nitrogen fertilizer and irrigation, reducing environmental pollution, maintaining soil microbial ecosystems, and achieving sustainable agricultural development.

## Materials and methods

### Experimental site

**Figure 5:**
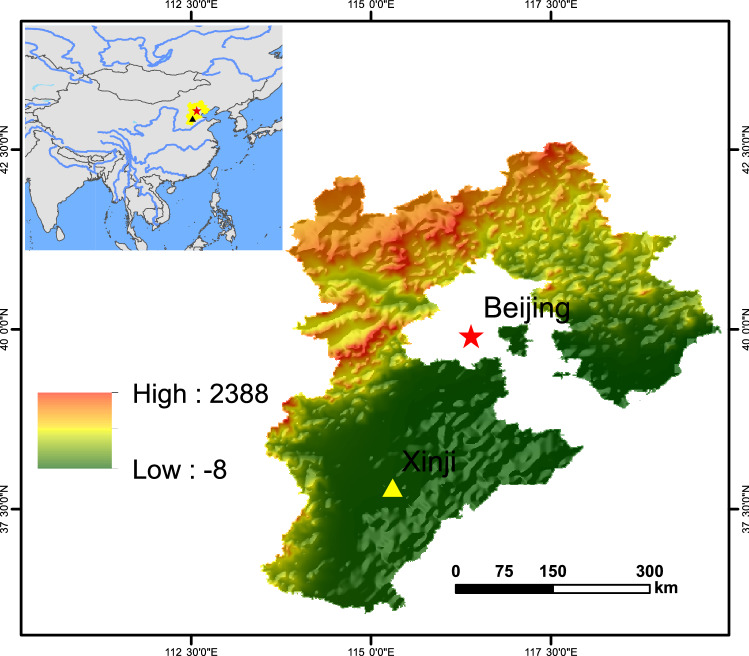
Location of Xinji experimental station. The height above sea level ranges from − 8 m (green) to 2388 m (red).

The study was conducted over three growing seasons for winter wheat from 2015–2017 at the Xinji Experimental Station (37°48′18'' N, 115°18′40'' E) of Hebei Agricultural University (Fig. 5). The typical cropping system is a winter wheat and summer maize double-cropping system. The area is semi-arid with a monsoon climate and an average annual temperature of 13 °C and average annual precipitation of 466.4 mm. More than 70% of the precipitation occurred from July to September. Monthly distributions of precipitation for 2015–2018 is shown in Table 4. According to the U.S. soil taxonomy, the soil was classified as an aquic inceptisol with a sandy loam texture. The chemical parameters of the soil at the start of the experiment were as follows: pH 7.86, bulk density (BD) 1.20 g cm^−3^, 18.70 g kg^−1^ soil organic carbon (SOC), 0.60 g kg^−1^ total nitrogen (TN), 37.05 mg kg^−1^ available phosphorus (AP), and 165.74 mg kg^−1^ available potassium (AK).

**Table 4:** Monthly distributions of precipitation (mm) for 2015–2018.

Year	Month												Total precipitation
	1	2	3	4	5	6	7	8	9	10	11	12	
2015	0	0	0	19.6	81.9	19.8	30.1	140.3	38.9	17.2	50.5	0	398.3
2016	1.5	13.7	0	10.3	18.2	78.4	164	56.4	35.9	52.3	0	0	430.7
2017	0	0	0	14.9	14.3	53.5	92.5	87.7	6.9	109	7	0	385.8
2018	1	0.4	14.4	98.6	55.2	29.3	82.4	186.8	6.6	6.4	1.2	7.3	489.6

### Experimental design

The experiments were designed as a split plot arrangement, with irrigation frequency (no irrigation, irrigation once at jointing, and irrigation twice at jointing and anthesis, expressed by W0, W1, and W2, respectively) as the main plots, and nitrogen fertilization rate (0, 120, and 240 kg ha^−1^, expressed by N0, N1, and N2, respectively) as the sub-plots. The amount of irrigation was 600 m^3^ ha^−1^ per time. The main plot size was 45 m × 17 m and the sub-plot was 15 m × 17 m, and each main plot was separated from each other by a 1 m zone. Each treatment combination was replicated three times. A local winter wheat (*Triticum aestivum* L.) cultivar, Jimai 585, was planted in the 2015–2016, 2016–2017, and 2017–2018 growth seasons.

Every year, comminuted maize straw was plowed as a base fertilizer, and the same amount of chemical fertilizer (135 kg ha^−1^ P_2_O_5_, and 150 kg ha^−1^ K_2_O) was applied before planting. Half of the nitrogen fertilizer (CH_4_N_2_O) in W1 and W2 was applied as a base fertilizer before planting, and half was applied at the jointing stage. All of the nitrogen fertilizers in W0 were applied as base before planting. The management practices for controlling pests, diseases, and weeds complied with local practices for agricultural production.

### Soil sampling and chemical analyses

In the 2018 harvest stage, which represented the 3rd year of the experiment, five soil samples were collected from the 0–10 cm soil layer in each treatment plot using a soil-drilling sampler. All five soil samples were composited and sieved through a 2-mm mesh to thoroughly homogenize and remove the roots, plant residues, and stones. A portion of each soil sample was collected in a 50-mL centrifuge tube, placed in an ice-box, and transferred to the laboratory. The tubes were archived at − 80 °C until DNA extraction. The physicochemical properties of the other soil samples were determined.

All analyses of soil properties were performed using routine methods^58^. Soil moisture content was measured by oven-drying the soil to a constant weight at 105 °C for 24 h. Soil pH values were determined by dilution (5:1) with a 0.01 M CaCl_2_ solution. Soil organic carbon (SOC) was measured using the K_2_Cr_2_O_7_ oxidation method. Total nitrogen (TN) was analyzed using the semi-Kjeldahl method. The available phosphorus (AP) content was first extracted from the soil in a 0.5 M NaHCO_3_ solution and then determined using the vanadium molybdate yellow colorimetric method^59^. The available potassium (AK) was extracted from the soil by ammonium acetate and ascertained by flame photometry (FP-6410; Xinyi Instruments Co. Ltd., Shanghai, China) as described by Bao^59^. The top soil bulk density (BD) was measured during field sampling with a 100 cm^3^ steel cylinder.

### DNA extraction and PCR amplification

We used the CTAB/SDS method to extract total genomic DNA from all the soil samples. DNA concentration and purity were measured using a 1% agarose gel. Depending on the concentration, DNA was diluted to 1 ng μL^−1^ using sterile water. The V4 regions of the 16S rRNA genes were amplified using specific primers: 515F (5’-GTGCCAGCMGCCGCGGTAA-3’) and 806R (5’-GGACTACHVGGGTWTCTAAT-3’). All PCR reactions were performed in 30 μL mixture with 15 μL of Phusion® High-Fidelity PCR Master Mix (New England Biolabs), 0.2 μM of forward and reverse primers, and about 10 ng of template DNA. The reactions were carried out in a thermal cycler (Bio-Rad Laboratories, Hercules, CA) using the following program: initial denaturation at 98 °C for 1 min, followed by 30 cycles of 98 °C for 10 s, annealing at 50 °C for 30 s, and elongation at 72 °C for 30 s; and finally, 72 °C for 5 min.

The obtained PCR products were mixed with the same volume of 1 × loading buffer (containing SYBR green) and were checked on 2% agarose gel to ascertain the specificity of 16 s rRNA gene amplification. The PCR products were mixed in equal density ratios and then purified using the GeneJETTM Gel Extraction Kit (Thermo Scientific). According to the manufacturer's instructions, the Ion Plus Fragment Library Kit (48 reactions, Thermo Scientific) was used to generate sequencing libraries. The library quality was assessed on a Qubit 2.0 Fluorometer (Thermo Scientific). Finally, the libraries were sequenced on the Ion S5™ XL platform, and 400 bp/600 bp single-end reads were generated.

### Bioinformatics and statistical analysis

According to the unique barcode, single-end reads were assigned to samples and truncated by cutting off the barcodes and primer sequences. With the Cut adapt (Version 1.9.1, http://cutadapt.readthedocs.io/en/stable/) quality-controlled process, the raw reads were filtered under specific filtering conditions to obtain high-quality clean reads. The reads were compared with the Sliva database (https://www.arb-silva.de/). The chimera sequences were detected using the UCHIME algorithm (http://www.drive5.com/usearch/manual/uchime_algo.html), and then removed^60^. Finally, high-quality clean reads were obtained. Sequence analysis was performed using the Uparse software (Version 7.0.1001, http://drive5.com/uparse/)^61^. High-quality sequences with ≥ 97% similarity were assigned to the same operational taxonomic units (OTUs). Representative OTU sequences were selected for further annotation. The Silva Database (https://www.arb-silva.de/) was used to annotate taxonomic information using the Mothur algorithm for each representative sequence^62^. To ascertain the differences in the dominant species in different samples and the phylogenetic relationship of different OTUs, the MUSCLE software (Version 3.8.31, http://www.drive5.com/muscle/) was used to conduct multiple sequence alignments^63^. The OTU abundance information of each sample was normalized to that of the sample with the least sequences. The original sequence obtained by sequencing was submitted to the NCBI database, and the sequence number was obtained: SRR13767216.

The complexity of species diversity for samples was analyzed using alpha diversity, including observed richness (Sobs), Chao1, Shannon, Shannon, and phylogenetic diversity (PD) indices. All the indices were calculated using the free online platform, Majorbio I-Sanger Cloud Platform (www.i-sanger.com). Unweighted pair-group method, while the arithmetic means (UPGMA) clustering was performed as a type of hierarchical clustering method to interpret the distance matrix using average linkage and was conducted using QIIME software (Version 1.7.0). The differences between groups in the beta diversity index were evaluated using principal coordination analysis (PCoA) based on the composition of the bacteria using the Bary-Curtis metrics by R software (Version 2.15.3). SPSS (Version 20.0) was used to perform ANOVA to calculate significant differences in the dominant microbial taxon composition, alpha diversity, and soil variables. The interactions among alpha diversity, relative abundance of bacteria (phylum, genus, and OTUs), and soil properties were confirmed through Pearson's correlation analysis using SPSS (Version 20.0).

The bacteria community features in the different treatments of irrigation and nitrogen fertilization were characterized using the linear discriminant analysis effect size (LEfSe) method (http://huttenhower.sph.harvard.edu/lefse/). LEFSe is an algorithm for high-dimensional biomarker discovery that emphasizes both statistical significance and biological relevance^64^. The non-parametric factorial Kruskal–Wallis sum rank test was used to identify the bacteria taxa with different treatments using LEfSe. Linear discriminant analysis (LDA) was used to estimate the effect of size on each differentially abundant feature. LDA > 3.5, and p < 0.05, were used for the biomarkers evaluated.

### Ethical statement

Wheat collection was approved by the local government and scientific research institutions.

The authors declare that the methods of our study are in full compliance with the relevant institutional, national, and international guidelines and legislation.

## Supplementary Information

Supplementary Information 1.
