# Supplementary material for: Irrigation has a higher impact on soil bacterial abundance, diversity and composition than nitrogen fertilization
Source: Sci Rep. 2021 Aug 19;11:16901. doi: 10.1038/s41598-021-96234-6 (PMC8377015; doi:10.1038/s41598-021-96234-6)
Supplement: Supplementary file 1 — Supplementary Information 1. [file 41598_2021_96234_MOESM1_ESM.docx]

**Supplementary Information**

**Irrigation has a higher impact on soil bacterial abundance, diversity and composition than nitrogen fertilization**

**Haoran Li****^1†^, Hongguang Wang^1†^, Bin Jia^1^, Dongxiao Li^1^, Qin Fang^1^, Ruiqi Li^1^***

**^1^**State Key Laboratory of North China Crop Improvement and Regulation/Key laboratory of Crop Growth Regulation of Hebei Province, College of Agronomy, Hebei Agricultural University, Baoding, China.

*** Correspondence:**

Ruiqi Li

liruiqi2019@126.com

^†^ These authors have contributed equally to this work.

College of Agronomy, Hebei Agricultural University, 2596#, Lekai South street, Baoding 071000, Hebei, China.

**Supplementary Figure S1.** A linear discriminant analysis effect size (LEfSe) method identifies the significantly different abundant taxa of bacteria in different treatments of irrigation frequency under N0 treatment. The taxa with the absolute LDA > 3.5 and p < 0.05 are shown. This figure is drawn by LEfSe (Version 1.0, http://huttenhower.sph.harvard.edu/galaxy/root?tool_id=lefse_upload) software.


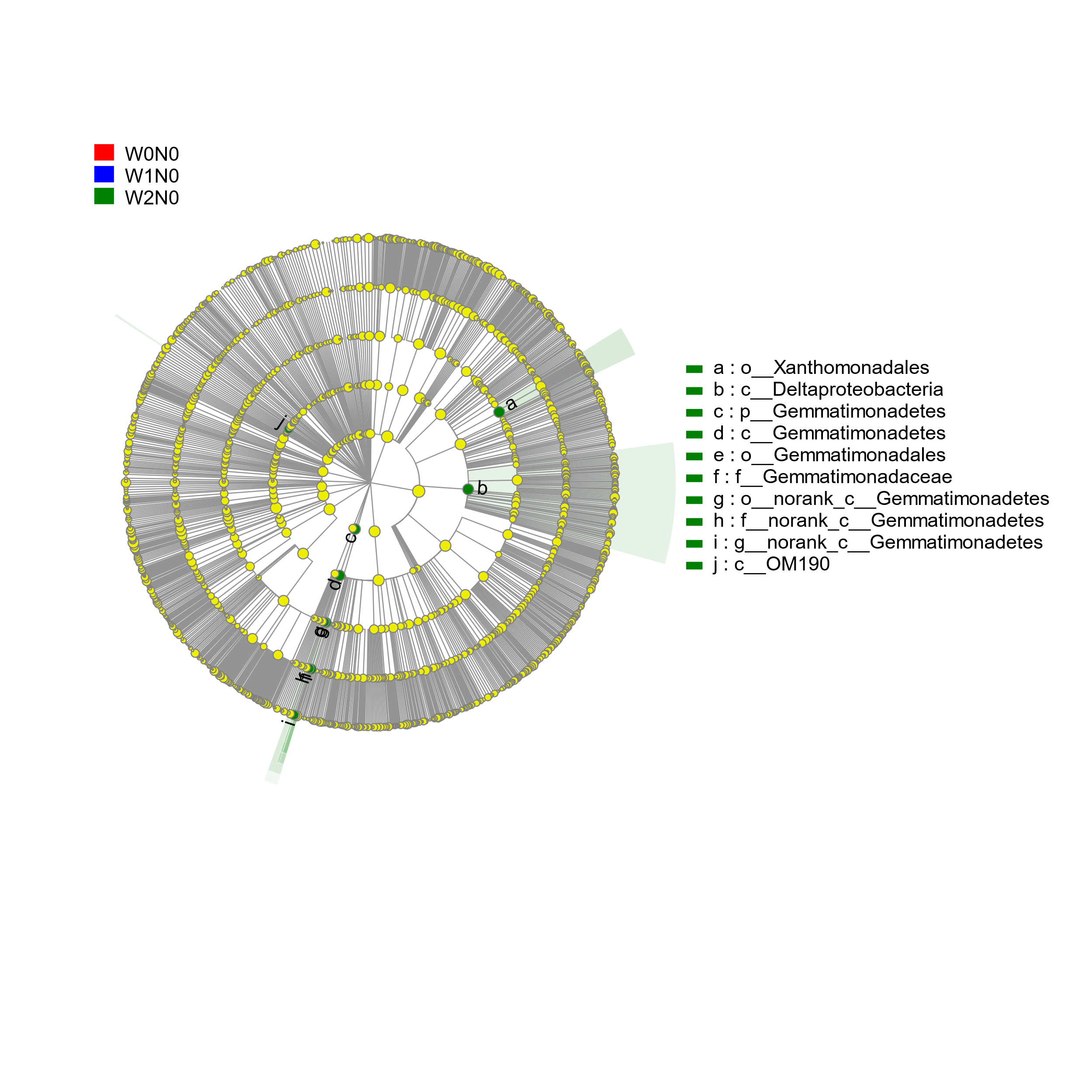


**Supplementary Figure S2.** A linear discriminant analysis effect size (LEfSe) method identifies the significantly different abundant taxa of bacteria in different treatments of irrigation frequency under N1 treatment. The taxa with the absolute LDA > 3.5 and p < 0.05 are shown. This figure is drawn by LEfSe (Version 1.0, http://huttenhower.sph.harvard.edu/galaxy/root?tool_id=lefse_upload) software.


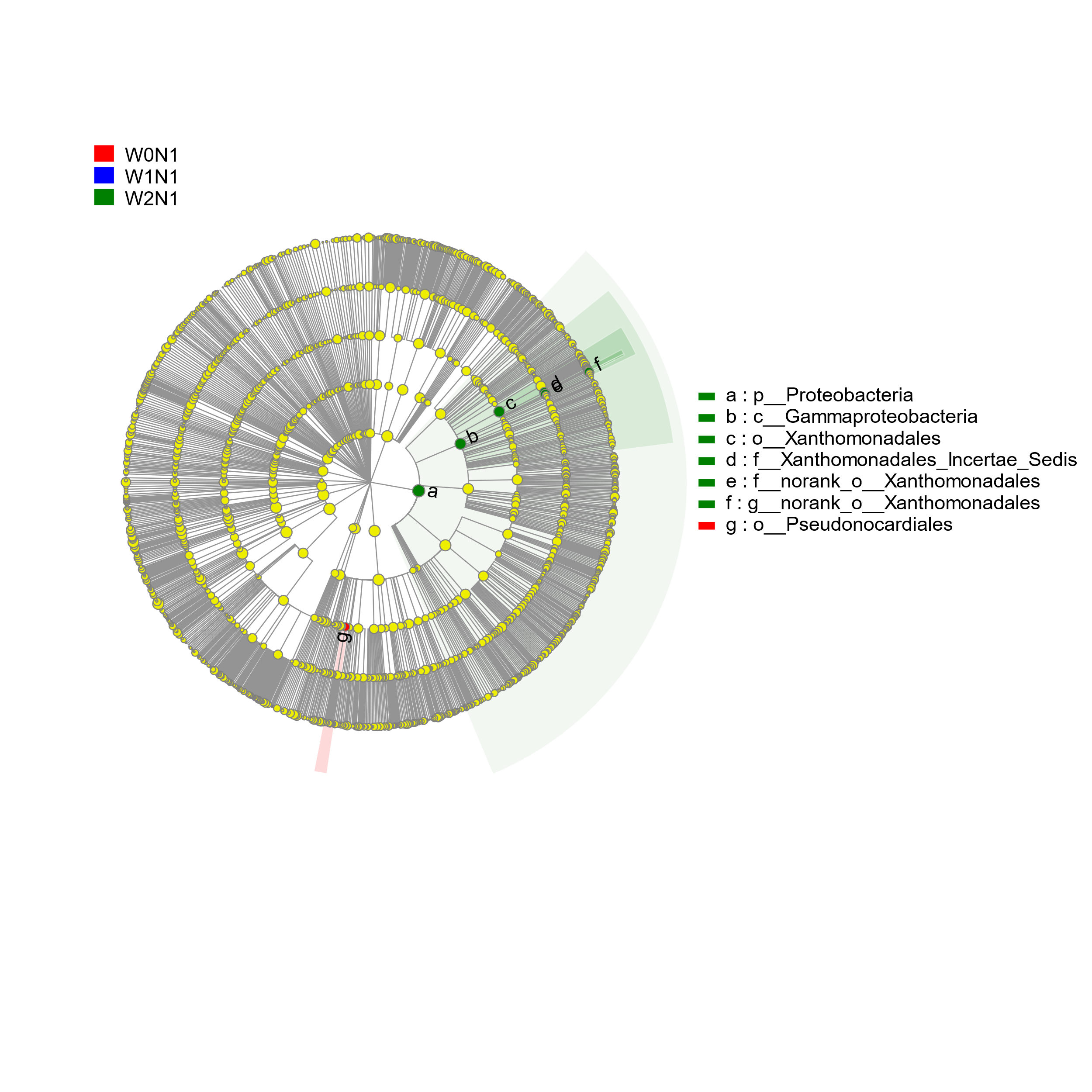


**Supplementary Figure S3.** A linear discriminant analysis effect size (LEfSe) method identifies the significantly different abundant taxa of bacteria in different treatments of irrigation frequency under N2 treatment. The taxa with the absolute LDA > 3.5 and p < 0.05 are shown. This figure is drawn by LEfSe (Version 1.0, http://huttenhower.sph.harvard.edu/galaxy/root?tool_id=lefse_upload) software.


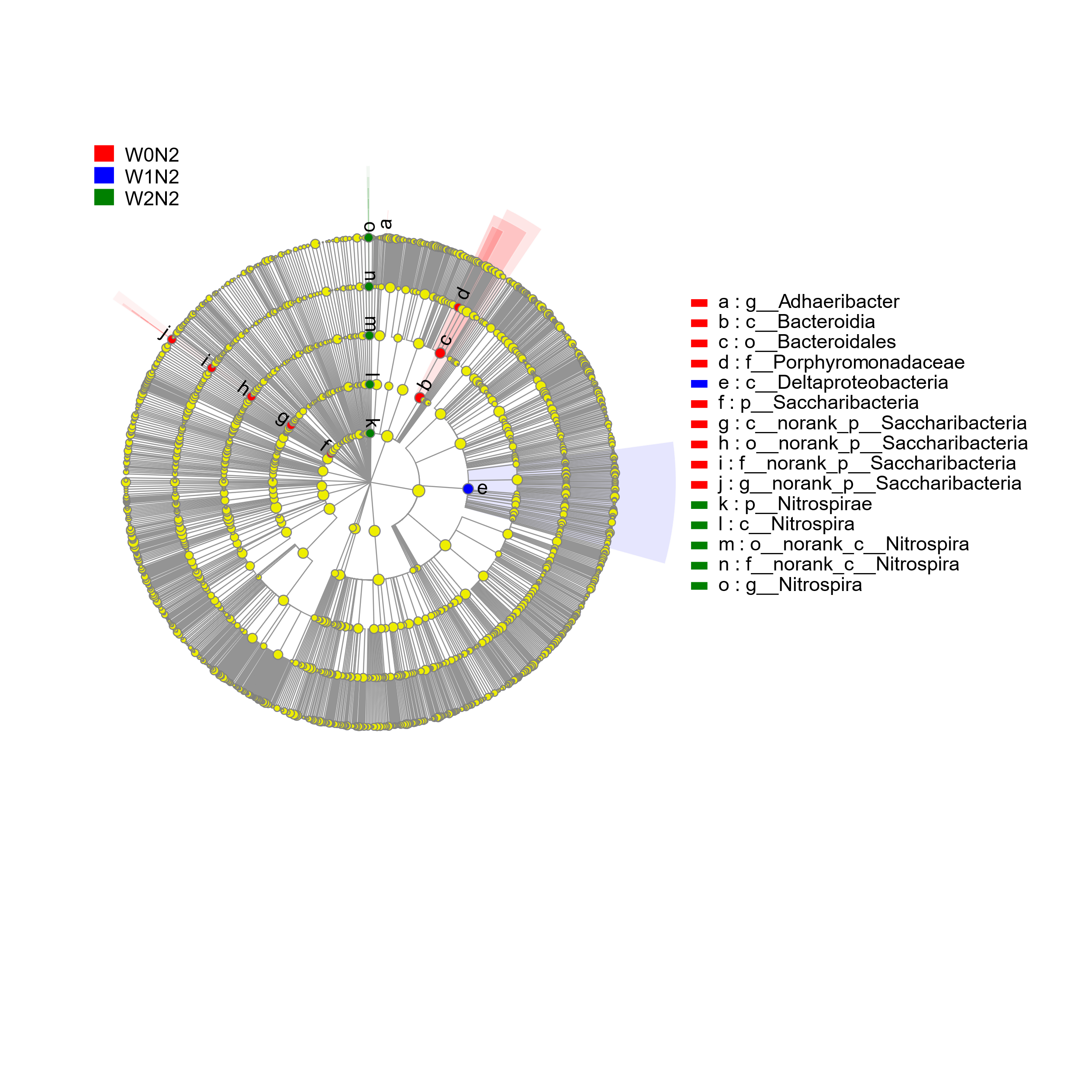


**Supplementary Figure S4.** A linear discriminant analysis effect size (LEfSe) method identifies the significantly different abundant taxa of bacteria in different treatments of nitrogen fertilization rate under W0 treatment. The taxa with the absolute LDA > 3.5 and p < 0.05 are shown. This figure is drawn by LEfSe (Version 1.0, http://huttenhower.sph.harvard.edu/galaxy/root?tool_id=lefse_upload) software.


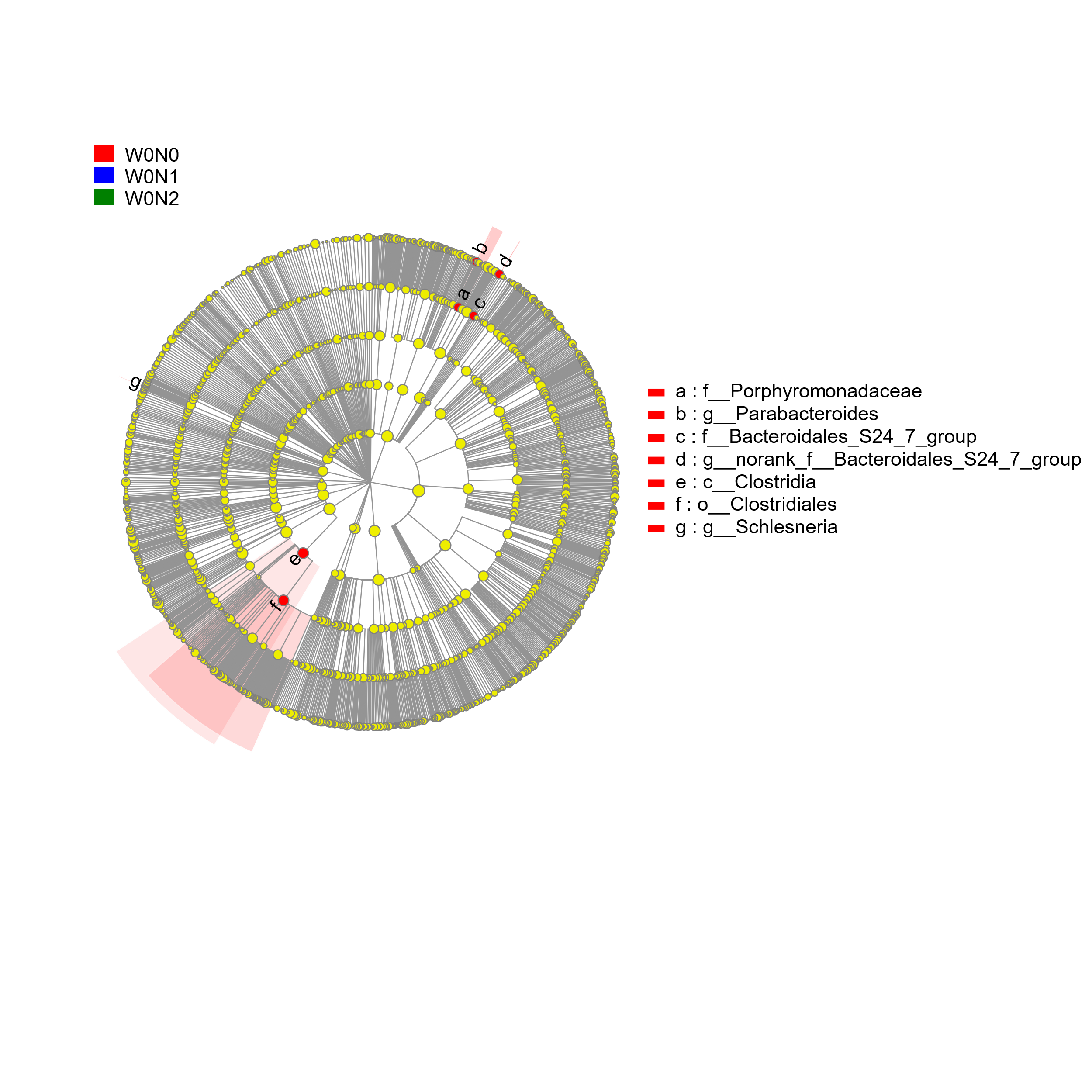


**Supplementary Figure S5.** A linear discriminant analysis effect size (LEfSe) method identifies the significantly different abundant taxa of bacteria in different treatments of nitrogen fertilization rate under W1 treatment. The taxa with the absolute LDA > 3.5 and p < 0.05 are shown. This figure is drawn by LEfSe (Version 1.0, http://huttenhower.sph.harvard.edu/galaxy/root?tool_id=lefse_upload) software.


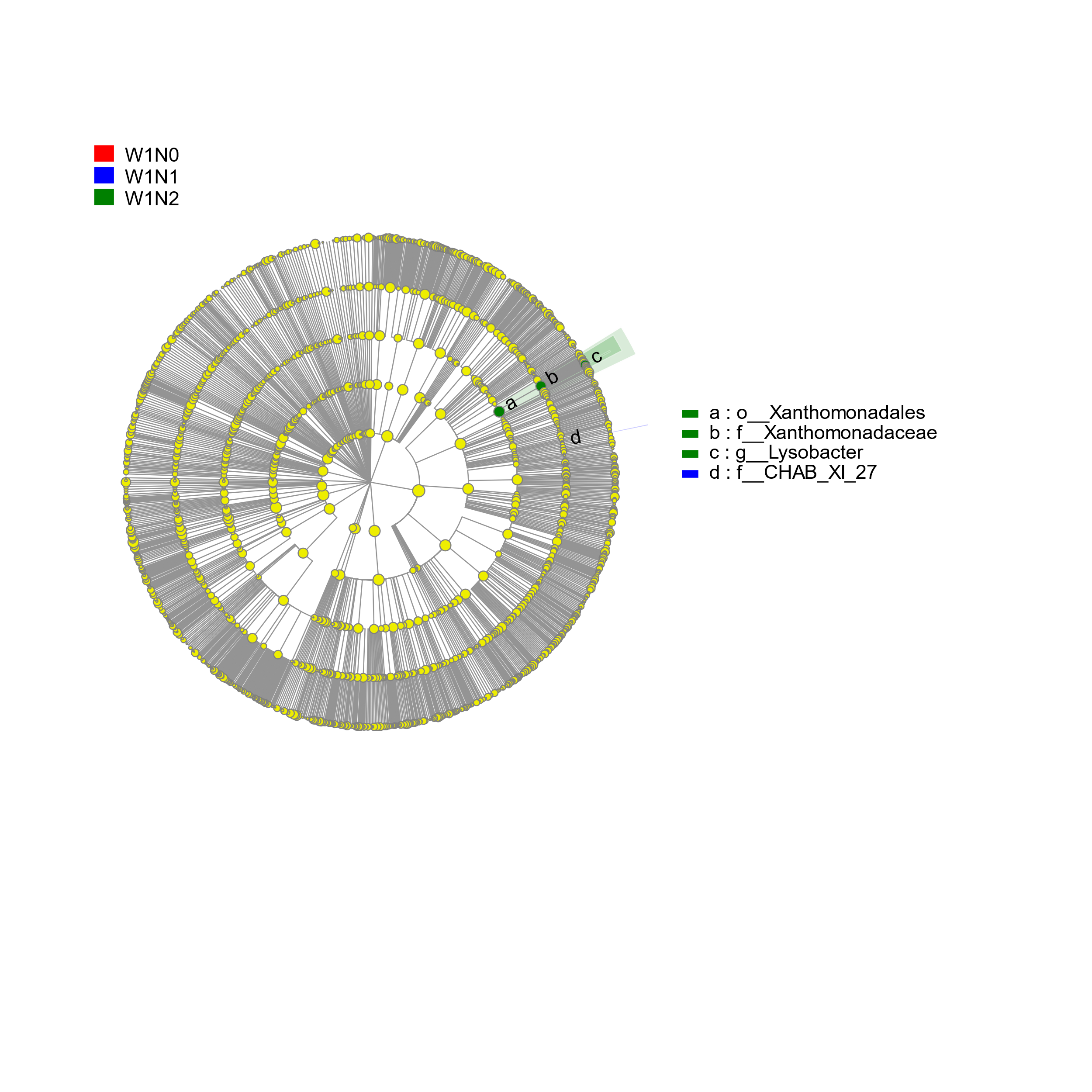


**Supplementary Figure S6.** A linear discriminant analysis effect size (LEfSe) method identifies the significantly different abundant taxa of bacteria in different treatments of nitrogen fertilization rate under W2 treatment. The taxa with the absolute LDA > 3.5 and p < 0.05 are shown. This figure is drawn by LEfSe (Version 1.0, http://huttenhower.sph.harvard.edu/galaxy/root?tool_id=lefse_upload) software.


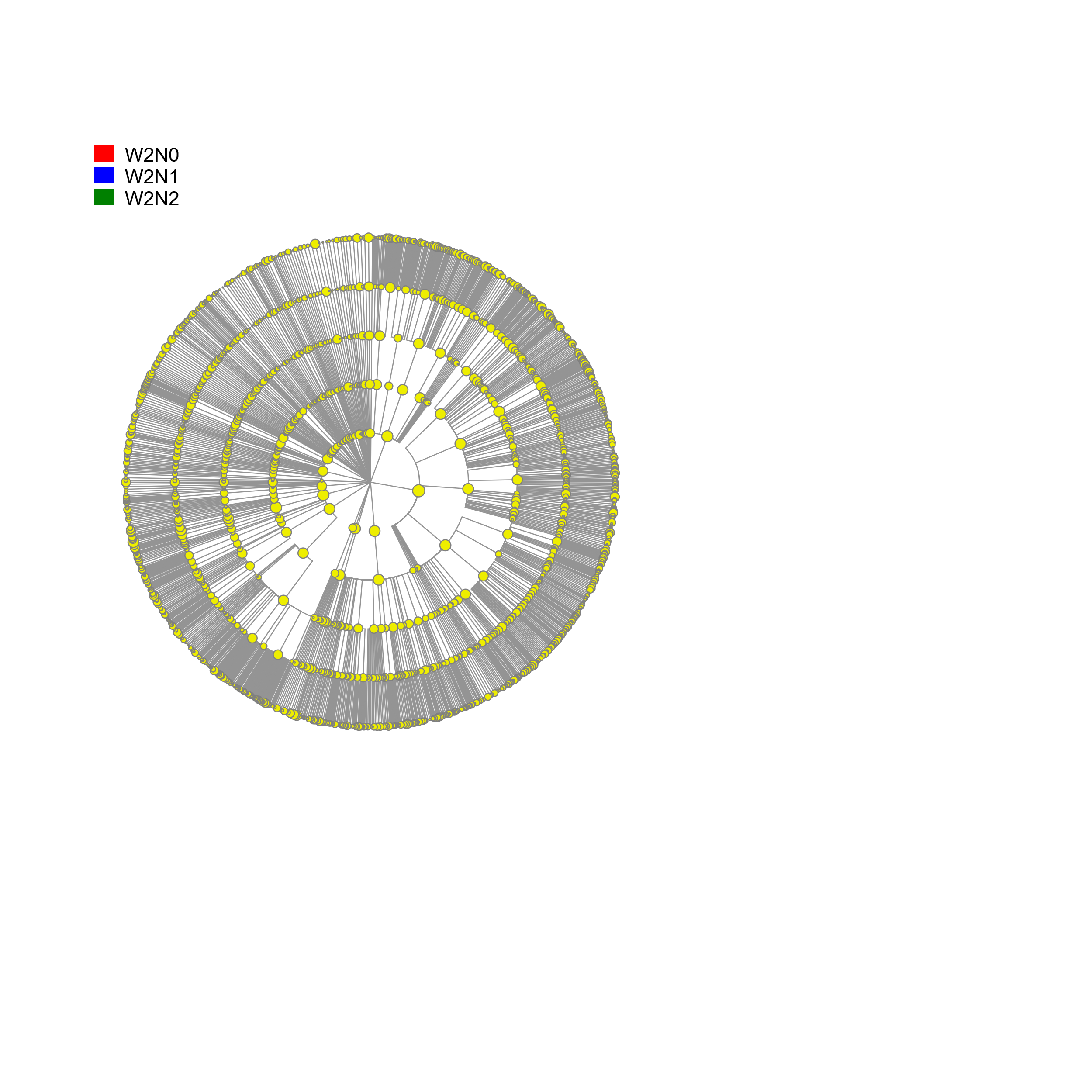


**Supplementary Table S1.** Effect of different treatments and the interaction between irrigation frequency and nitrogen fertilization rate on the relative abundance (%) of the dominant OTUs. NS, not significant (p > 0.05). * p < 0.05 significant levels. ** p < 0.01 significant levels. *** p < 0.001 significant levels. Different letters within the same column denote significant differences (p < 0.05) among soils.

| Treatment | OTU82 | OTU95 | OTU96 | OTU1428 | OTU1740 | OTU1827 | OTU2822 | OTU2894 | OTU6387 | OTU6495 |
| --- | --- | --- | --- | --- | --- | --- | --- | --- | --- | --- |
| W0N0 | 0.79±0.07 | 0.46±0.10 | 0.27±0.06 | 0.43±0.08 | 0.28±0.03 | 0.17±0.02 | 0.74±0.05 | 0.31±0.03 | 0.23±0.06 | 0.27±0.10 |
| W0N1 | 0.81±0.16 | 0.34±0.05 | 0.23±0.09 | 0.71±0.05 | 0.24±0.08 | 0.09±0.05 | 0.47±0.10 | 0.19±0.07 | 0.15±0.04 | 0.25±0.11 |
| W0N2 | 0.98±0.28 | 0.48±0.05 | 0.35±0.08 | 0.60±0.18 | 0.31±0.08 | 0.15±0.05 | 0.63±0.11 | 0.31±0.09 | 0.29±0.04 | 0.28±0.07 |
| W1N0 | 0.99±0.14 | 0.57±0.09 | 0.38±0.06 | 0.67±0.08 | 0.41±0.06 | 0.30±0.07 | 0.70±0.12 | 0.40±0.03 | 0.31±0.06 | 0.26±0.09 |
| W1N1 | 1.17±0.12 | 0.71±0.14 | 0.46±0.04 | 0.57±0.06 | 0.48±0.13 | 0.33±0.04 | 0.78±0.04 | 0.32±0.09 | 0.40±0.04 | 0.26±0.05 |
| W1N2 | 0.92±0.12 | 0.93±0.13 | 0.44±0.01 | 0.41±0.06 | 0.43±0.10 | 0.29±0.03 | 0.93±0.06 | 0.43±0.07 | 0.46±0.03 | 0.26±0.03 |
| W2N0 | 0.93±0.10 | 0.80±0.13 | 0.48±0.04 | 0.55±0.02 | 0.68±0.19 | 0.42±0.15 | 1.20±0.17 | 0.51±0.13 | 0.46±0.04 | 0.40±0.16 |
| W2N1 | 0.77±0.18 | 0.78±0.08 | 0.38±0.06 | 0.45±0.04 | 0.56±0.12 | 0.38±0.04 | 0.99±0.03 | 0.43±0.08 | 0.49±0.07 | 0.34±0.13 |
| W2N2 | 0.63±0.06 | 0.69±0.09 | 0.43±0.03 | 0.35±0.12 | 0.44±0.04 | 0.32±0.07 | 0.80±0.11 | 0.28±0.07 | 0.40±0.08 | 0.34±0.08 |
| Irrigation frequency |  |  |  |  |  |  |  |  |  |  |
| W0 | 0.86±0.19a | 0.43±0.09b | 0.28±0.09b | 0.58±0.16a | 0.28±0.07c | 0.14±0.05c | 0.61±0.14c | 0.27±0.08b | 0.22±0.07c | 0.27±0.08a |
| W1 | 1.02±0.16a | 0.73±0.19a | 0.43±0.05a | 0.55±0.13a | 0.44±0.09b | 0.31±0.04b | 0.80±0.12b | 0.38±0.07a | 0.39±0.08b | 0.26±0.05a |
| W2 | 0.78±0.17a | 0.76±0.10a | 0.43±0.06a | 0.45±0.11b | 0.56±0.16a | 0.37±0.10a | 1.00±0.20a | 0.41±0.14a | 0.45±0.07a | 0.36±0.11a |
| Nitrogen fertilization rate |  |  |  |  |  |  |  |  |  |  |
| N0 | 0.90±0.13a | 0.61±0.18a | 0.38±0.10a | 0.55±0.12a | 0.46±0.21a | 0.30±0.14a | 0.88±0.27a | 0.41±0.11a | 0.33±0.11a | 0.31±0.12a |
| N1 | 0.92±0.23a | 0.61±0.22a | 0.36±0.11a | 0.58±0.12a | 0.43±0.18a | 0.27±0.14a | 0.75±0.23a | 0.32±0.12a | 0.35±0.16a | 0.29±0.10a |
| N2 | 0.84±0.22a | 0.70±0.21a | 0.41±0.06a | 0.46±0.16a | 0.39±0.09a | 0.25±0.09a | 0.79±0.15a | 0.34±0.10a | 0.38±0.09a | 0.29±0.06a |
| Two-way ANOVA |  |  |  |  |  |  |  |  |  |  |
| W | NS | ** | ** | * | * | ** | ** | ** | * | NS |
| N | NS | NS | NS | NS | NS | NS | NS | NS | NS | NS |
| W×N | NS | ** | NS | * | NS | NS | ** | NS | *** | NS |

**Supplementary Table S2.** Correlation analysis among alpha diversity, relative abundance of dominant bacteria (phylum, genus and OTU) and soil properties. * p < 0.05 significant levels. ** p < 0.01 significant levels. *** p < 0.001 significant levels.

| Parameter | pH | Moisture | TN | SOC | C/N ratio | AP | AK | BD | Yield |
| --- | --- | --- | --- | --- | --- | --- | --- | --- | --- |
| **Soil properties** |  |  |  |  |  |  |  |  |  |
| pH |  | -0.829** | 0.129 | 0.099 | -0.137 | -0.699* | -0.697* | -0.427 | -0.549 |
| Moisture |  |  | -0.189 | -0.198 | 0.216 | 0.760* | 0.720* | 0.340 | 0.309 |
| TN |  |  |  | 0.923*** | -0.993*** | 0.137 | 0.141 | -0.378 | 0.653 |
| SOC |  |  |  |  | -0.942*** | -0.023 | 0.107 | -0.126 | 0.763* |
| C/N ratio |  |  |  |  |  | -0.063 | -0.137 | 0.389 | -0.675* |
| AP |  |  |  |  |  |  | 0.717* | 0.053 | 0.371 |
| AK |  |  |  |  |  |  |  | -0.110 | 0.552 |
| BD |  |  |  |  |  |  |  |  | 0.144 |
| **Alpha diversity** |  |  |  |  |  |  |  |  |  |
| Sobs | -0.896** | 0.664 | -0.177 | -0.257 | 0.180 | 0.643 | 0.673* | 0.083 | 0.377 |
| Shannon | -0.840** | 0.654 | 0.008 | -0.071 | 0.019 | 0.798* | 0.803** | 0.034 | 0.497 |
| Chao1 | -0.881** | 0.725* | -0.093 | -0.209 | 0.101 | 0.694* | 0.754* | -0.003 | 0.390 |
| Shannon even | -0.828** | 0.633 | -0.002 | -0.081 | 0.026 | 0.785* | 0.794* | 0.010 | 0.487 |
| PD | -0.955*** | 0.733* | -0.195 | -0.205 | 0.201 | 0.700* | 0.702* | 0.258 | 0.461 |
| **Phylum** |  |  |  |  |  |  |  |  |  |
| Proteobacteria | -0.885** | 0.878** | 0.035 | 0.004 | -0.942*** | -0.023 | 0.717* | 0.053 | 0.371 |
| Bacteroidetes | 0.848** | -0.766* | 0.095 | 0.049 | -0.125 | -0.673* | -0.703* | -0.523 | -0.474 |
| Actinobacteria | 0.658 | -0.768* | 0.039 | 0.122 | -0.045 | -0.649 | -0.677* | 0.119 | -0.303 |
| Acidobacteria | -0.039 | 0.106 | -0.523 | -0.361 | 0.547 | -0.085 | -0.016 | 0.621 | -0.275 |
| Gemmatimonadetes | -0.861** | 0.936*** | -0.128 | -0.211 | 0.161 | 0.819** | 0.833** | 0.168 | 0.333 |
| Firmicutes | -0.642 | 0.428 | 0.265 | 0.273 | -0.252 | 0.563 | 0.418 | 0.298 | 0.680* |
| Planctomycetes | -0.768* | 0.540 | -0.281 | -0.163 | 0.305 | 0.494 | 0.532 | 0.623 | 0.378 |
| Chloroflexi | -0.866** | 0.621 | -0.014 | 0.100 | 0.009 | 0.457 | 0.412 | 0.637 | 0.681* |
| Verrucomicrobia | 0.476 | -0.649 | -0.211 | -0.190 | 0.239 | -0.355 | -0.429 | 0.037 | -0.409 |
| Nitrospirae | -0.932*** | 0.901*** | -0.246 | -0.202 | 0.245 | 0.659 | 0.826** | 0.331 | 0.430 |
| **Genus** |  |  |  |  |  |  |  |  |  |
| Norank-c-Acidobacteria | 0.416 | 0.700* | -0.212 | -0.141 | 0.230 | 0.586 | 0.726* | 0.407 | 0.416 |
| Norank-f-Nitrosomonadaceae | -0.856** | 0.883** | -0.103 | -0.178 | 0.113 | 0.767* | 0.897** | 0.017 | 0.390 |
| Norank-c-OPB35 soil group | 0.055 | -0.252 | -0.433 | -0.392 | 0.460 | -0.118 | -0.124 | 0.204 | -0.304 |
| Norank-f-Gemmatimonadaceae | -0.721* | 0.751* | -0.233 | -0.285 | 0.274 | 0.769* | 0.826** | 0.127 | 0.227 |
| Sphingomonas | -0.236 | 0.578 | 0.436 | 0.391 | -0.431 | 0.394 | 0.424 | -0.077 | 0.385 |
| RB41 | 0.239 | -0.040 | -0.532 | -0.398 | 0.559 | -0.224 | -0.180 | 0.442 | -0.489 |
| Norank-c-Gemmatimonadetes | -0.738* | 0.872** | -0.239 | -0.366 | 0.266 | 0.776* | 0.833** | -0.043 | 0.144 |
| Haliangium | -0.802** | 0.850** | -0.276 | -0.319 | 0.283 | 0.703* | 0.871** | 0.039 | 0.258 |
| Lysobacter | -0.829** | 0.831** | -0.065 | -0.105 | 0.087 | 0.763* | 0.921*** | 0.153 | 0.432 |
| Norank-o-Xanthomonadales | -0.879** | 0.837** | 0.012 | 0.039 | -0.004 | 0.776* | 0.919*** | 0.174 | 0.607 |
| **OTU** |  |  |  |  |  |  |  |  |  |
| OTU82 | 0.061 | -0.241 | -0.171 | -0.139 | 0.192 | -0.025 | 0.132 | 0.005 | -0.083 |
| OTU95 | -0.793* | 0.648 | 0.136 | 0.080 | -0.135 | 0.726* | 0.939*** | -0.076 | 0.602 |
| OTU96 | -0.814** | 0.659 | 0.086 | 0.006 | -0.058 | 0.722* | 0.821** | 0.079 | 0.520 |
| OTU1428 | 0.491 | -0.342 | -0.336 | -0.208 | 0.364 | -0.403 | -0.426 | 0.362 | -0.493 |
| OTU1740 | -0.811** | 0.875** | -0.256 | -0.242 | 0.259 | 0.627 | 0.864** | 0.195 | 0.317 |
| OTU1827 | -0.935*** | 0.881** | -0.251 | -0.236 | 0.260 | 0.710* | 0.827** | 0.295 | 0.405 |
| OTU2822 | -0.706* | 0.788* | -0.212 | -0.295 | 0.207 | 0.627 | 0.879** | -0.143 | 0.217 |
| OTU2894 | -0.587 | 0.707* | -0.322 | -0.432 | 0.355 | 0.739* | 0.815** | -0.104 | 0.027 |
| OTU6387 | -0.843** | 0.779* | 0.119 | 0.097 | -0.114 | 0.776* | 0.960*** | 0.016 | 0.685* |
| OTU6495 | -0.556 | 0.851** | -0.142 | -0.203 | 0.144 | 0.476 | 0.600 | 0.029 | 0.124 |
